# Histopathological Evaluation of PYGO2 Expression in Esophageal Squamous Cell Carcinoma

**DOI:** 10.30699/ijp.2024.2024609.3269

**Published:** 2024-10-02

**Authors:** Sima Ardalan Khales, Habibeh Rahmani kalat, Sedigheh Soleymani, Amir Hossein Jafarian, Mohammad Mahdi Forghanifard

**Affiliations:** 1 *Immunology Research Center, Mashhad University of Medical Sciences, Mashhad, Iran*; 2 *Department of Biology, Damghan Branch, Islamic Azad University, Damghan, Iran*; 3 *Department of Pathology, Ghaem Hospital, Mashhad University of Medical Sciences, Mashhad, Iran*

**Keywords:** Esophageal squamous cell carcinoma, Immunohistochemistry, PYGO2, Wnt/β-catenin signaling pathway

## Abstract

**Background & Objective:**

Esophageal squamous cell carcinoma (ESCC) is one of the world's deadliest cancer diseases. Deregulation of developmental signaling pathways such as Wnt/β-catenin is frequently implicated in a wide range of human cancers. The present study was designed to analyze the expression of the Pygopus2 (PYGO2) protein, the main co-activator of the Wnt/β-catenin signaling pathway, in ESCC tissues and evaluate its probable correlation with clinicopathological features of patients.

**Methods:**

In this study, PYGO2 protein expression was assessed in tumors and margin normal tissues from 50 ESCC patients using immunohistochemical analysis, and its clinicopathological relevance in the patients was evaluated.

**Results:**

A significant PYGO2 overexpression was observed in %32 of the tumor cells. Interestingly, PYGO2 expression was significantly correlated with the depth of tumor invasion (*P*= 0.021).

**Conclusion:**

PYGO2 protein may be highly expressed in ESCC in correlation with the invasiveness of the disease. Therefore, it may be used as a biomarker for diagnosis of invasive ESCC and a putative therapeutic target to inhibit ESCC invasiveness.

## Introduction

Esophageal squamous cell carcinoma (ESCC) is among the leading causes of cancer-related death in the world, with more than 544,000 deaths annually (1). ESCC has a high prevalence in Asian countries and accounts for more than 90% of esophageal cancers (2, 3). ESCC is the second most frequent cancer in Iran, which has high morbidity and mortality rates (4, 5). Despite advances in ESCC prognoses, this fatal disease has poor diagnoses, with survival rates of less than 20% in developed countries and less than 5% in most developing countries (1). Consequently, it makes sense to investigate the underlying molecular mechanisms of ESCC development to identify precise and effective targets. 

Deregulation of the Wnt/β-catenin signaling pathway leads to the development and progression of different malignancies, such as ESCC (6, 7). The Wnt/β-catenin signaling pathway plays a critical role in cellular/developmental processes and tissue homeostasis (8), as well as cancer formation. 

Pygopus family PHD finger 2(PYGO2), a crucial co-activator of the Wnt/β-catenin transcriptional complex (9), has been detected in multiple tumors, including colon adenocarcinoma (COAD) (10), epithelial ovarian (11), renal cell carcinoma (12), and breast (13) cancers. Nonetheless, the association between PYGO2 gene expression and ESCC is somewhat controversial. Due to the importance of the role of the PYGO2 signaling pathway in tumorigenesis, our aim in this study was to compare PYGO2 protein expression in esophageal normal and tumor tissues and evaluate its possible correlations with ESCC progression.

## Materials and Methods

### Study Population

In this study, PYGO2 protein expression in 50 ESCC cases who had not received any treatment before surgery was assessed using immunohistochemical staining. The Cochran method was used to determine the sample size (14). The samples with the criteria considered were collected between 2015 and 2020 and included in the study. The clinicopathological information was obtained from each patient record at Ghaem Hospital, including a sample ID number, age, gender, tumor size, and stage. The ethics committee of Mashhad University of Medical Sciences approved the study, and all the patients signed informed consent. The histopathological characteristics such as grade of differentiation, lymph node metastasis (LNM), depth of tumor invasion (T), and the surgical stage were determined according to the Union International Cancer TNM classification guidelines (15). 

### Immunohistochemistry (IHC)

Fifty formalin-fixed paraffin-embedded ESCC and margin normal tissue blocks were collected from Ghaem Hospital. Then, each paraffin-embedded block was sectioned with a microtome in 4 to 5μm thick sections. Then, to adhere to the tissue and cell components, the slides were placed in the oven at 70 to 80°C for 5 min. Next, the sections were dewaxed and rehydrated in a xylene-ethanol series. For robust results with heat-induced antigen retrieval (HIER), slides were placed in the antigen retrieval solution (Tris/EDTA PH 6-7) (ab93684, Abcam, Cambridge, MA, USA) and boiled for 20 min at 98°C, which antigens were retrieved. After heat treatment, the slides were cooled at RT and washed in phosphate-buffered saline (PBS). The sections were then treated with 0.3% hydrogen peroxide (H2O2) (Sigma-Aldrich; Merck KGaA, Darmstadt, Germany) in methanol for 30 min at RT and subsequently incubated with a blocking solution counting 100–400 µl of BSA (RE7102, Thermo Fisher Scientific, Waltham, MA, USA) for 1 h at RT, following which the sections were washed three times with dH2O for 5 min. After endogenous peroxidase blocking, rabbit anti-PYGO2 monoclonal primary antibody (1:50) (EPR2024, Abcam, Cambridge, MA, USA) was used for 1 hr at 4℃. After two washes in PBS for 5 min, each section was incubated for 20 min in the HRP-conjugated secondary antibody (#8114, Cell Signaling Technology, Danvers, MA, USA) at RT. Then, the peroxidase reactivity was developed by 100–400 µl DAB (chromogen 3,3′-diaminobenzidine) reagent (RE7105, Leica- NovoCastra, Newcastle, UK) to each tissue section for 10 min, and after washing with water, slides were counterstained with Mayer’s hematoxylin for 30 seconds. Next, washing in running tap water for 5 min, the samples were dehydrated in ethanol solution (75%, 85%, 95%, and 100%) for 1 min in each concentration and immersed in xylene before mounting, and observed by light microscope (Olympus, USA). PYGO2 test staining was independently repeated three times. The ESCC tissue samples treated without primary antibodies were used as a negative control. Each section was blindly reviewed and scored by a pathologist using light microscopy.

### Immunohistochemical Scoring

The immunohistochemical scoring system was used to assess the IHC staining steps in cancer samples (16). The intensity of IHC staining for PYGO2 and the proportion of stained cells were evaluated. Stained cells were scored as 0 (no staining), 1 (weak, diffuse cytoplasmic staining or stronger intensity in <10% of tumor cells), 2 (moderate or strong staining in 10–50%), 3 (moderate or strong staining in 50–80%), and 4 (strong staining in ≥80%). Tumors with a mean score of 0 or 1 were considered negative, whereas those with scores 2–4 were regarded as positive. The Intensity staining (IS) score was measured for all images. The formula for calculating the IS score was as follows: the percentage of positive cells (P) x the intensity (I). A total score was assessed as the percentage of positive cells multiplied by the staining intensity (range 0 - 12). 

### Statistical Analysis

All statistical analyses were performed by SPSS software 19.9 (SPSS, Chicago, IL. USA). Independent sample t-test and ANOVA were used to examine probable correlations between PYGO2 protein expression and clinicopathological factors. P-values of <0.05 were considered to demonstrate a statistically significant difference.

## Results

### Study Population

Fifty ESCC patients (21 males and 29 females, ages ranging from 17 to 84 years with a mean age of 55.32 ± 15.84 years) were enrolled in this study. The size of the specimens ranged from 1.5 to 12 cm. Most of the tumor samples were in tumor growth stages II or III; two were in stage I. Based on the histopathological analyses, 5 tumor samples were in Grade I, while 41 and 4 were in Grade II and III, respectively. Furthermore, 50.8% of the tumors metastasized to the lymph nodes. The clinical and pathological characteristics of the patients are summarized in Table 1.

### Levels of PYGO2 Expression in ESCC Patients

**Fig. 1:**
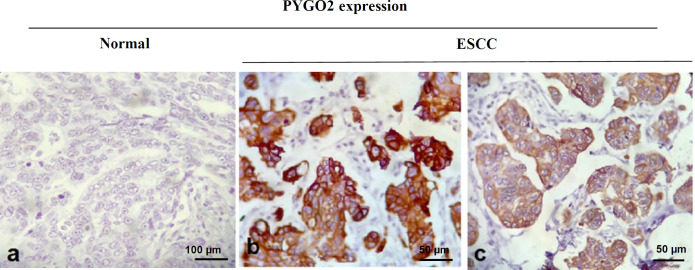
Immunohistochemical staining patterns of PYGO2 protein expression in normal and ESCC tissues. (a) PYGO2 expression in the esophageal normal tissues. Scale bar = 100 μm, (b) High expression of PYGO2 in the ESCC tissues. Scale bar = 50 μm, (c) Low expression of PYGO2 in the ESCC tissues. Scale bar = 50 μm.

**Fig. 2:**
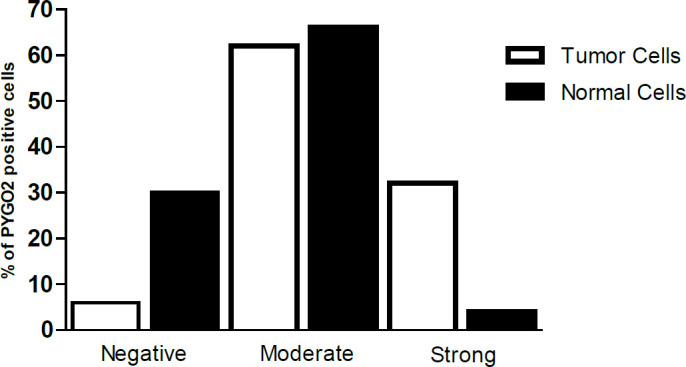
The percentages of PYGO2-positive cells in the ESCC tissues. The expression level of PYGO2 protein differed between the tumors and their corresponding normal tissues. Thirty three (66%) out of 50 normal samples, showed moderate PYGO2 expression in the ESCC tissues, 15 (30%) showed negative PYGO2 expression, and only 2 (4%) showed strong expression of PYGO2 in the ESCC tissues.

Levels of PYGO2 expression were compared in the tumors with their corresponding normal margins using IHC staining. The PYGO2 IHC staining images of the normal and ESCC tissues are illustrated in Figure 1. As shown in Table 2, PYGO2 protein expression in the tumors was significantly higher than in the normal cells (32%). Furthermore, 33 (66%) out of 50 normal samples, showed moderate PYGO2 expression in the ESCC tissues, 15 (30%) showed negative PYGO2 expression, and only 2 (4%) showed strong expression of PYGO2 in the ESCC tissues (Table 2, Figure 2).

**Fig. 3:**
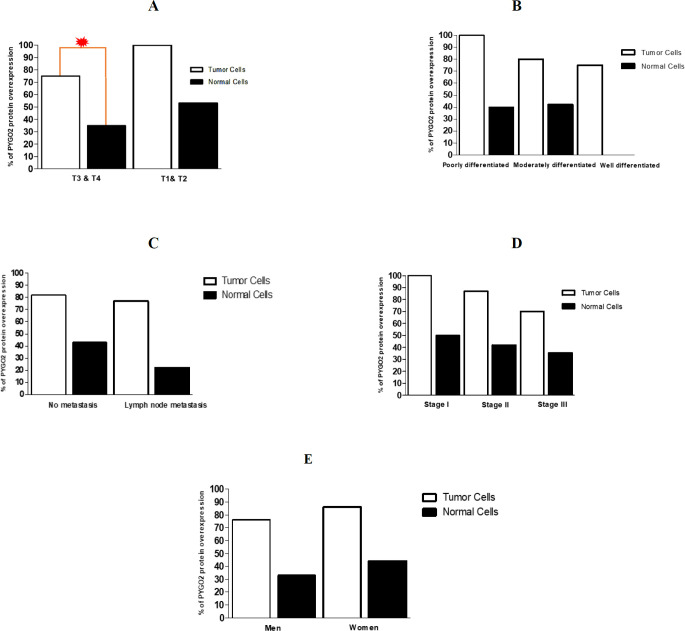
Association of overexpression of PYGO2 protein and clinicopathological features of the ESCC patients. (A) Invasive tumor samples (T3, T4) were characterized by a significantly higher expression of PYGO2 protein compared to other samples. No significant association between overexpression of PYGO2 protein and other clinico-pathological factors such as (B) grade of differentiation, (C) metastasis, (D) stages of cancer and (E) sex.

Using statistical analysis, a significant association was observed between PYGO2 expression and depth of the tumor invasion (*P*=0.021), Figure 3a. According to our observations, samples with tumor invasion (T3 & T4) showed a significantly higher expression of PYGO2 protein than other samples. Indeed, 28 out of the 37 (75.67%) invasive tumor samples (T3, T4) showed overexpression of PYGO2 protein, whereas 13 tumor samples without invasion to adventitia (T2) showed PYGO2 overexpression.

We did not find any significant association between overexpression of PYGO2 and other clinico-pathological factors such as sex, metastasis, tumor grades, and cancer stages (Figure 3b-f, Table 3).

## Discussion

In this study, we found that the PYGO2 protein is significantly overexpressed in ESCC in correlation with the depth of tumor invasion. This finding may support the hypothesis that PYGO2 can be a new molecular marker of invasiveness in ESCC. Since PYGO2 plays a vital role in cancer progression and development through the wnt signaling pathway, it can be suggested as a probable therapeutic target to inhibit and reverse cancer invasiveness.

 PYGO2 ablation restored the chemotherapeutic drug sensitivity in breast-resistant cells and repressed breast cancer cell growth. (17). In hepatocellular carcinoma (HCC), Wnt/β-catenin and Myc signaling are upregulated, and the correlative overexpression of nuclear PYGO2 and Myc represent the malignant characteristics of HCC (18). In addition to the in vivo and in vitro functional assays, the clinical expression analysis discovered that high PYGO2 expression acts as an oncogenic driver of prostate cancer, which overexpression of PYGO2 protein was related to LNM and bone metastasis, high-grade cancer, and biochemical recurrence (BCR) (19). PYGO2 plays a critical function in regulating the transcription process, as it can modulate multidrug resistance (MDR) transcription by binding to the MDR1 promoter sequence and inducing MDR1 activation in gastric cancer. It has been reported that PYGO2 could be a new biomarker for detecting drug resistance through overexpression of MDR1 in gastric cancer (20). In glioma cancer cells, in addition to overexpression of PYGO2 in the tumor cells, an association between the PYGO2 overexpression and the tumor grade was also reported. It has been shown that inhibiting PYGO2 protein expression prevents cell proliferation in glioma (21). Repression of PYGO2 inhibited cancer development, aggression, and epithelial-mesenchymal transition (EMT) (22). Suppression of PYGO2 mRNA can inhibit the expression of Wnt/β-catenin signaling target genes in colorectal cancer (23). Popadiuk *et al.* showed that suppressing PYGO2 expression in ovarian cancer cell lines impedes tumor growth, highlighting the importance of PYGO2 protein in carcinogenesis pathways (24). These results support the hypothesis that inhibition of PYGO2 expression may prevent cancer development. 

Consistent with these findings, our investigation of ESCC cases showed that the expression of PYGO2 was significantly higher in the ESCC tumors compared to the normal samples. Furthermore, in line with a previous report on glioma cancer, the rate of tumor grade increased with PYGO2 overexpression, and a positive correlation between PYGO2 expression and tumor grade was observed (21). Interestingly, it has been shown that PYGO2 mRNA expression has a significant association with tumor grade and size in ESCC (25). In our study, a similar observation was indicated. A significant association between PYGO2 protein expression and tumor invasion was found.

Although automated immunohistochemical platforms have significantly enhanced the reproducibility and reliability of IHC assays, mainly in the clinical environment, manual IHC technique still affords greater flexibility, provides a specific antibody-antigen interaction optimization, and improves results, mainly in the research setting. Accordingly, the PYG02 tests staining were independently repeated three times, and the results were reproducible and statistically significant (*P*<0.05).

Finally, our findings showed the important role of PYGO2 in the progression of ESCC and suggest that PYGO2 may potentially serve as a diagnostic marker for ESCC. 

**Table 1:** The Patient’s clinico-pathological characteristics.

Variables		Number
Patients (Mean age 55.32 yrs)		50
	
Gender	Male	21
	Female	29
Tumor grade	PD	5
	MD	41
	WD	4
Lymph node metastasis	Yes	29
	No	21
Tumor stage	I	2
	II	31
	III	17
Depth of tumor invasion	T1	0
	T2	13
	T3	35
	T4	2

PD Poorly differentiated; MD Moderately differentiated; WD Well differentiated

Patients without invasion to adventitia (T1, T2); Patients with invasion of tumor to adventitia (T3, 4)

**Table 2:** PYGO2 protein expression in the tumors in comparison to the normal cells.

The expression level of PYGO2 protein	Tumor Cells		Normal Cells	
	Number of patients	Percent (%)	Number of patients	Percent (%)
Negative	3	6	15	30
Moderate	31	62	33	66
Strong	16	32	2	4
Total	**50**	**100**	**50**	**100**

**Table 3:** Relationship between expression of PYGO2 protein in the ESCC tumor and normal tissue, clinicopathological characteristics and P-values.

**Characteristic**	**Demographic**	**Expression of PYGO2 Protein in Tumor Cells**			**Expression of PYGO2 protein in normal cells**			**P-value**
		Negative (n)	Low (n)	High (n)	Negative (n)	Low (n)	High (n)	
Sex	Men	2	3	16	8	6	7	0.222
	Women	1	3	25	7	9	13	
Grade of differentiation	P.D	0	0	5	2	1	2	0.842
	M.D	3	5	33	12	11	18	
	W.D	0	1	3	1	3	0	
Depth of tumor invasion	T1 & T2	0	0	13	3	3	7	0.021*
	T3 & T4	3	6	28	12	12	13	
Lymph node metastasis	No metastasis	2	5	34	13	10	18	0.882
	Lymph node metastasis	1	1	7	2	5	2	
Stage of tumor progression	I	0	0	2	1	0	1	0.146
	II	1	3	27	9	9	13	
	III	2	3	12	5	6	6	

(n) number of cases.

*Correlation is significant at the 0.05 level.

## Conclusion

Our results revealed significant overexpression of PYGO2 protein in the ESCC samples and supported the hypothesis that PYGO2 can be a new molecular marker of invasiveness in patients with ESCC. A significant correlation between expression of this protein and patients’ clinicopathological features, including tumor invasion was also found. It is believed that PYGO2 may play a vital role in cancer progression; therefore, it can be used as a therapeutic target for cancer therapy.
